# Community Structure and Function of High-Temperature Chlorophototrophic Microbial Mats Inhabiting Diverse Geothermal Environments

**DOI:** 10.3389/fmicb.2013.00106

**Published:** 2013-06-03

**Authors:** Christian G. Klatt, William P. Inskeep, Markus J. Herrgard, Zackary J. Jay, Douglas B. Rusch, Susannah G. Tringe, M. Niki Parenteau, David M. Ward, Sarah M. Boomer, Donald A. Bryant, Scott R. Miller

**Affiliations:** ^1^Department of Land Resources and Environmental Sciences, Montana State University, Bozeman, MT, USA; ^2^Thermal Biology Institute, Montana State University, Bozeman, MT, USA; ^3^Novo Nordisk Foundation Center for Biosustainability, Technical University of Denmark, Hørsholm, Denmark; ^4^Center for Genomics and Bioinformatics, Indiana University, Bloomington, IN, USA; ^5^Department of Energy Joint Genome Institute, Walnut Creek, CA, USA; ^6^Search for Extraterrestrial Intelligence Institute, Mountain View, CA, USA; ^7^National Aeronautics and Space Administration Ames Research Center, Mountain View, CA, USA; ^8^Western Oregon University, Monmouth, OR, USA; ^9^Department of Biochemistry and Molecular Biology, The Pennsylvania State University, University Park, PA, USA; ^10^Department of Chemistry and Biochemistry, Montana State University, Bozeman, MT, USA; ^11^Department of Biological Sciences, University of Montana, Missoula, MT, USA

**Keywords:** microbial mats, microbial interactions, phototrophic bacteria, functional genomics, thermophilic bacteria

## Abstract

Six phototrophic microbial mat communities from different geothermal springs (YNP) were studied using metagenome sequencing and geochemical analyses. The primary goals of this work were to determine differences in community composition of high-temperature phototrophic mats distributed across the Yellowstone geothermal ecosystem, and to identify metabolic attributes of predominant organisms present in these communities that may correlate with environmental attributes important in niche differentiation. Random shotgun metagenome sequences from six phototrophic communities (average ∼53 Mbp/site) were subjected to multiple taxonomic, phylogenetic, and functional analyses. All methods, including G + C content distribution, MEGAN analyses, and oligonucleotide frequency-based clustering, provided strong support for the dominant community members present in each site. Cyanobacteria were only observed in non-sulfidic sites; *de novo* assemblies were obtained for *Synechococcus-*like populations at *Chocolate Pots* (CP_7) and *Fischerella*-like populations at *White Creek* (WC_6). Chloroflexi-like sequences (esp. *Roseiflexus* and/or *Chloroflexus* spp.) were observed in all six samples and contained genes involved in bacteriochlorophyll biosynthesis and the 3-hydroxypropionate carbon fixation pathway. Other major sequence assemblies were obtained for a Chlorobiales population from CP_7 (proposed family *Thermochlorobacteriaceae*), and an anoxygenic, sulfur-oxidizing *Thermochromatium*-like (Gamma-proteobacteria) population from *Bath Lake Vista Annex* (BLVA_20). Additional sequence coverage is necessary to establish more complete assemblies of other novel bacteria in these sites (e.g., Bacteroidetes and Firmicutes); however, current assemblies suggested that several of these organisms play important roles in heterotrophic and fermentative metabolisms. Definitive linkages were established between several of the dominant phylotypes present in these habitats and important functional processes such as photosynthesis, carbon fixation, sulfur oxidation, and fermentation.

## Introduction

Many naturally occurring microorganisms have eluded isolation, due in part to a poor understanding of the chemical, physical, and biotic factors defining their realized niches (Rappé and Giovannoni, 2003). Moreover, much of the sequence diversity revealed by amplification of specific gene targets (e.g., 16S rRNA) is susceptible to biases inherent in primer-design and PCR protocols. Random shotgun sequencing of environmental DNA provides a direct and potentially less biased view of the composition and functional attributes of microbial communities. For example, three new chlorophototrophic organisms (i.e., organisms capable of (bacterio)chlorophyll-based phototrophy) were discovered in prior metagenome analyses of oxygenic mats in YNP, two of which lie outside the clades of known phototrophic organisms in the Chlorobiales and Chloroflexi (Klatt et al., 2011). Moreover, the third organism, “*Candidatus* Chloracidobacterium thermophilum” (“*Ca*. C. thermophilum”), represents the only known occurrence of chlorophototrophy in the phylum Acidobacteria (Bryant et al., 2007; Klatt et al., 2011; Garcia Costas et al., 2012). Metagenome sequencing and subsequent bioinformatic analyses provide an opportunity to identify the metabolic attributes of uncultivated organisms that can be used to postulate detailed biochemical linkages among individual community members necessary for the development of computational models describing microbial interaction and community function (Taffs et al., 2009).

High-temperature phototrophic microbial mats have served as models for studying microbial community structure and function. Studies have included investigations of microbial community composition (Miller et al., 2009), the ecophysiology of novel isolates (Pierson and Castenholz, 1974; Bryant et al., 2007; van der Meer et al., 2010), comparative genomics, metagenomics, and metatranscriptomics (Bhaya et al., 2007; Klatt et al., 2007, 2011; Becraft et al., 2011; Liu et al., 2011, 2012; Melendrez et al., 2011), community network modeling (Taffs et al., 2009), phage-host interactions (Heidelberg et al., 2009), as well as theoretical models of evolution (Ward et al., 2008). The high temperature and relative geochemical stability of geothermal phototrophic mats in YNP generally result in communities with several dominant phylotypes and have provided opportunities for understanding environmental factors controlling community composition (Brock, 1978; Cohen and Rosenberg, 1989; Ward and Castenholz, 2000; Ward et al., 2012). Prior investigations have revealed that temperature, pH, and sulfide are among the most important environmental variables dictating differences in phototrophic mat community structure (Castenholz, 1976, 1977; Castenholz and Pierson, 1995; Madigan et al., 2005; Cox et al., 2011; Boyd et al., 2012). The presence of sulfide was used in the current study to separate anoxygenic versus oxygenic communities common in YNP (Inskeep et al., 2013). Oxygenic and/or anoxygenic photoautotrophs are generally the predominant primary producers in geothermal mats at temperatures of ∼50–72°C and moderately acidic to alkaline pH (5–9). These mat communities support a diverse array of (photo-) heterotrophic, fermentative, sulfate-respiring, and methanogenic organisms, whose physiological attributes are critical for understanding community function (Zeikus and Wolfe, 1972; Jackson et al., 1973; Henry et al., 1994; Nold and Ward, 1996; Ward et al., 1998; Taffs et al., 2009; Klatt et al., 2011; Liu et al., 2012).

The distribution of different chlorophototrophic bacteria is often controlled by specific geochemical parameters. For example, members of the Cyanobacteria are not generally found in acidic or sulfidic environments (Castenholz, 1976, 1977). However, filamentous anoxygenic phototrophs (FAPs) of the phylum Chloroflexi exhibit a wider habitat range than other chlorophototrophs. Closely related members of the Chloroflexi [>97% nucleotide identity (NT ID) of the 16S rRNA gene] with different phenotypes have been cultured from geothermal environments (Madigan et al., 1974; Madigan and Brock, 1975). FAPs isolated from a high-sulfide (>100 μM) spring in the absence of cyanobacteria (*Chloroflexus* sp. GCF strains) fixed inorganic carbon using sulfide as the electron donor (Giovannoni et al., 1987). However, most other cultured *Chloroflexus* spp. from low-sulfide environments are photoheterotrophic and do not utilize reduced sulfur for photosynthesis (Madigan et al., 1974; Pierson and Castenholz, 1974). Natural populations of FAPs are known to consume organic compounds produced by cyanobacterial community members (van der Meer et al., 2005); however, genomic and biochemical evidence is needed to improve our understanding of how different populations of Chloroflexi function *in situ*.

The overall goal of this study was to investigate the underlying environmental factors and potential physiological adaptations important in defining the microbial community structure and function of different types of chlorophototrophic mats commonly found in association with certain geothermal features of YNP (Inskeep et al., 2013). The specific objectives of this study were to (i) utilize metagenome sequencing and bioinformatic analyses to determine the community composition of thermal chlorophototrophic mats in YNP, (ii) identify key metabolic attributes of the major chlorophototrophic organisms present in these communities, and (iii) evaluate the predominant environmental and/or geochemical attributes that contribute to niche differentiation of thermophilic chlorophototrophic communities. The habitats sampled in the current study were chosen to focus on several of the major high-temperature phototrophic mat types that are distributed across the YNP geothermal ecosystem.

## Results

### Geochemical and physical context

**Figure 1:**
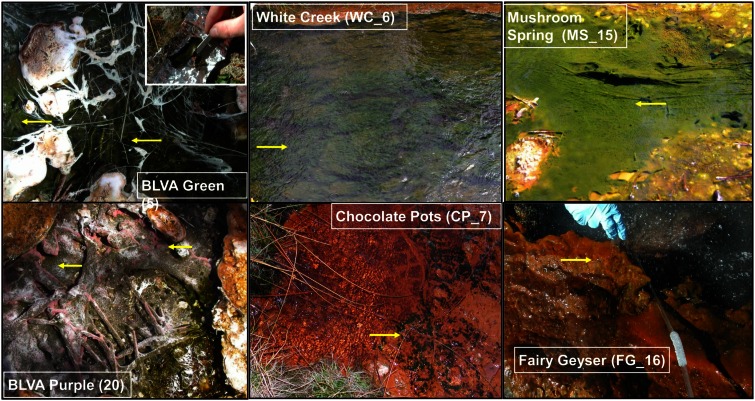
**Site photographs of phototrophic microbial mats selected for metagenome sequencing**. The sites cover a range in geochemical conditions including (i) highly sulfidic environments at *Bath Lake Vista Annex* (BLVA_5, 20), (ii) oxygenic phototrophic communities at *White Creek* (WC_6) and *Chocolate Pots* (CP_7), and (iii) subsurface mat layers at *Mushroom Spring* (MS_15) and *Fairy Geyser* (FG_16) (also oxygenic systems). The anoxygenic phototrophic communities at *Bath Lake Vista Annex* (BLVA) were sampled at two different time points (Table S2 in Inskeep et al., 2013) to compare *Chloroflexus* mats in the absence (BLVA_5) and presence (BLVA_20) of purple-bacteria (Arrows indicate approximate sample locations and types; inset at BLVA_5 shows mat dissection at sampling).

The predominant differences among the six phototrophic microbial mat communities included both geochemical characteristics such as pH and dissolved sulfide (DS), as well as temperature, and the sample depth (Figure 1; Table 1). Temperature ranged from 40–60°C across these six sites, and is a critical parameter controlling community composition. Four of the geothermal sites contained no measurable DS, while both samples from *Bath Lake Vista Annex Spring* (BLVA_5 and BLVA_20) were collected from hypoxic sulfidic environments (total DS ∼117 μM). Although the dissolved oxygen content at the source of *Chocolate Pots* (near sample location CP_7) was below detection (<1 μM), this spring contained no sulfide and high concentrations of Fe (II) (∼76 μM) (Table 1), which results in the precipitation of Fe(III)-oxides upon discharge and reaction with oxygen (Trouwborst et al., 2007). The phototrophic mat obtained from *White Creek* (WC_6) occurs within an oxygenated, alkaline-siliceous geothermal drainage channel containing no detectable DS (Table 1). The site was included in the study to target a population of the heterocyst-forming cyanobacterium *Fischerella* (*Mastigocladus*) *laminosus* that has been the focus of prior work at this location (Miller et al., 2006, 2007, 2009).

**Table 1:** **Sample locations and aqueous geochemical parameters^1^ of six, high-temperature phototrophic microbial communities sampled in Yellowstone National Park (YNP) and used for metagenome sequencing**.

Location	T (°C)	pH	Na^+^	Cl^−^	${\text{SO}}_{\text{4}}^{\text{2-}}$	^1^DIC	^1^DS	^1^DO	^1^DOC	As	^2^Fe	^3^${\text{NH}}_{\text{4}}^{\text{+}}$	Coordinates
	
	
			mM				μM						
Bath Lake Vista Annex-Green (BVLA_5)	57	6.2	3.9	4.4	5.6	15.8	117	<3	104	24	0.7	40	44° 57′54.180″N 110° 42′42.228″W
Bath Lake Vista Annex-Purple (BLVA_20)	54	6.2	5.5	5.7	7.3	24.2	117	<3	75	23	0.7	40	44° 57′54.180″N 110° 42′42.228″W
White Creek (WC_6)	52	8.2	3.6	1.8	0.23	nd	<3	188	nd	5	1.7	1.9	44° 31′53.399″N 110° 47′51.799″W
Chocolate Pots (CP_7)	52	6.2	4.1	0.89	0.23	13.2	<3	<3	38	9	75.5	4.2	44° 42′36.288″N 110° 44′28.824″W
Mushroom Spring (MS_15)	60	8.2	12.6	7.3	0.18	2.1	<3	141	nd	26	<1	4.4	44° 32′19.284″N 110° 47′52.692″W
Fairy Geyser (FG_16)	36–38	9.1	9.4	5.2	0.18	4.8	<3	31	30	13	<1	1.3	44° 32′31.812″N 110° 51′40.788″W
^4^Correlation (*r*^2^)		0.89^*^		0.93^*^	0.96^*^		0.99^***^				0.72^**^	0.99^**^	

*^1^DS, total dissolved sulfide; DO, dissolved oxygen; DIC, dissolved inorganic carbon; DOC, dissolved organic carbon*.

*^2^Mn (total soluble) values were also significant in CP_7 (24 μM) and WC_6 (5 μM), but low in other sites (0.1–0.2 μM, or below detection of 0.1 μM)*.

*^3^Nitrate values ranged from 2.1–6.7 μM across sites*.

^4^Correlation significance values: *p < 0.05, **p < 0.01, ***p < 0.001.

Samples from *Mushroom Spring* (MS_15) and *Fairy Geyser* (FG_16) were obtained from laminated phototrophic mats after removal of the top layer (See Materials and Methods). Dissection of these mats was performed to focus on FAPs, which were known to occur in higher abundance at greater depths below a surface layer dominated by cyanobacteria (Boomer et al., 2002; Nübel et al., 2002). The phototrophic mats at FG_16 are referred to as “splash-mats” due to the fact that these communities receive frequent inputs of geothermal water emanating from the main source pool (85–88°C) (Figure 1). The “splash-mats” surrounding FG_16 are reasonably thick (∼3–5 cm), and the sample discussed here was collected from a 2–4 mm “red-layer,” found within a temperature range of 35–50°C and a pH approaching 9 (Boomer et al., 2000, 2002). The visual characteristic of the “red-layer” was apparent during sampling and represents a different subsurface environment than the sample obtained from MS_15. No measurable DS was present in the bulk aqueous phase (Table 1) of these mats; however, subsurface mats in these systems (MS_15 and FG_16) have been shown to be less oxic than their respective near-surface layers (Jensen et al., 2011).

### Analysis of metagenome sequences

Individual sequences (average length ∼800 bp) were analyzed using several complementary approaches including alignment-based comparisons to reference databases, and evaluation of the guanine and cytosine content (% G + C) of each sequence read. In addition, comparison of all sequences to the NCBI nr database (blastx) was accomplished using MEGAN (Huson et al., 2007). The most highly represented phyla across all sites included the Chloroflexi (28%), Cyanobacteria (12%), Proteobacteria (8%), Bacteroidetes (6%), and Chlorobi (2%). Many sequence reads (27%) did not match those available in NCBI (“no hits”); this indicated that some members of these communities are not represented in current databases.

**Figure 2:**
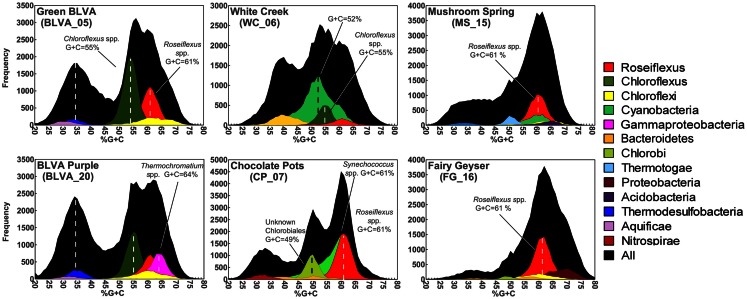
**Percent G + C content and taxonomic analysis of random shotgun sequence reads obtained from six thermophilic phototrophic mat communities from Yellowstone National Park (YNP)**. The frequency plot of all sequence reads (black) versus G + C content (%) is shown with corresponding taxonomic analysis (MEGAN-“blastx”) as indicated by the color key (right).

Taxonomic assignment of individual sequences was combined with %G + C distribution to obtain a profile of community composition (Figure 2). Each site contained populations similar to *Chloroflexus* and/or *Roseiflexus* spp., with average G + C contents of 55 and 61%, respectively. The two sulfidic samples (BLVA_5 and BLVA_20) showed contributions from both *Chloroflexus* and *Roseiflexus*-like populations (Figure 2). The oxic community from *White Creek* (WC_6) also contained significant contributions from *Chloroflexus*-like organisms, while CP_7, MS_15 and FG_16 were enriched in *Roseiflexus*-like sequences (Figure 2). All sites contain a significant number of sequences contributed from novel Chloroflexi that have not been adequately characterized, and for which appropriate reference organisms have not yet been cultivated or sequenced.

**Figure A1:**
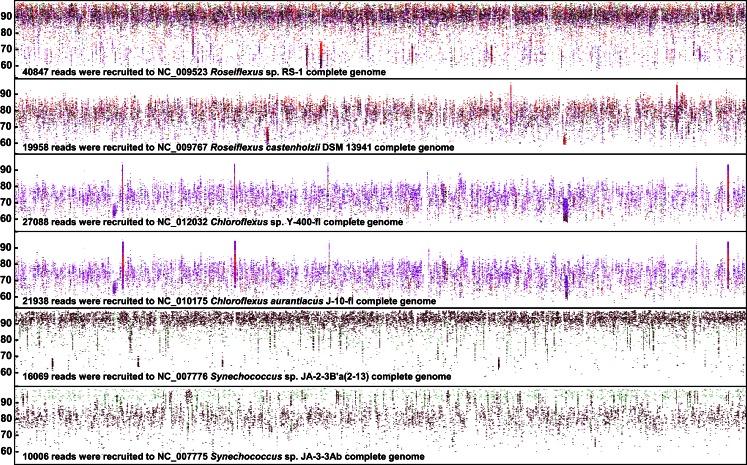
**Fragment recruitment of metagenome sequence reads from six phototrophic mat samples to the most relevant reference genomes (*y*-axis = 50–100% (nt); *x*-axis = genome position) currently available in reference databases**. Site Colors: *White Creek* (WC_6) = blue; *Chocolate Pots* (CP_7) = brown; *Mushroom Spring* (MS_15) = green; *Fairy Geyser* (FG_16) = red; *Bath Lake Vista Annex* (BLVA_5 and 20) = light and dark purple, respectively. Reference genomes: *Synechococcus* sp. B′ (JA-2-3B′a) and A (JA-3-3Ab); *Chloroflexus* sp Y-400-fl and *C. aurantiacus* J-10-fl; *Roseiflexus* sp RS-1 and *R. castenholzii* DSM 13941 (Plot constructed using JCVI bioinformatic utilities; Rusch et al., 2007).

**Figure A2:**
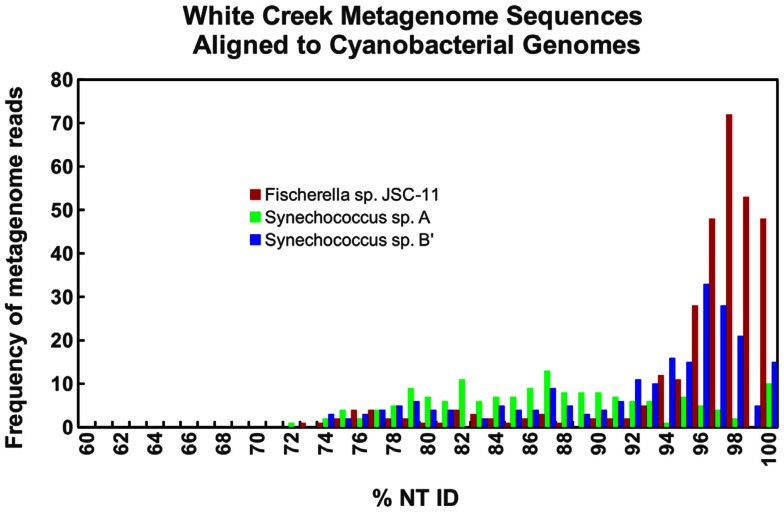
**BLASTN recruitment of White Creek (WC_6) metagenome sequence reads to representative cyanobacterial genomes**.

The phototrophic mat communities from WC_6 and CP_7 contained a significant fraction of sequences (23 and 25%, respectively) contributed from members of the Cyanobacteria. Both sites contained sequences related to *Synechococcus* spp. strains A and B′ (mean G + C content of 60%; Bhaya et al., 2007) (Figure 2; Figure A1 in Appendix), but the WC_6 community yielded a large proportion of Cyanobacteria-like sequences (73%) that could not be classified beyond the phylum-level, and these sequences exhibit a large range in G + C content (40–65%). *Fisherella laminosus* (order Stigonematales) has been shown to be an important community member at WC_6 (Miller et al., 2009), and many of the cyanobacterial sequences from WC_6 showed high sequence identity (95% average NT ID of alignments) to the draft genome of *Fischerella* sp. JSC-11 (average G + C = 41%; Figure A2 in Appendix), which was the only representative genome available for this group of cyanobacteria (at time of writing). The G + C content frequency plots also revealed major contributions from organisms within the Chlorobi (at sites CP_7 and FG_16), *Thermotoga* (MS_15), and *Thermochromatium* spp. (purple-sulfur bacteria) in BLVA_20 with an average G + C content of 64%. Moreover, all sites contained bacterial sequences that could not be identified beyond the level of Domain *Bacteria* (especially G + C contents ranging from 20–40%, Figure 2), in part because appropriate reference genomes are not currently available, and significant assemblies were not obtained for phylotypes present in lower abundance.

### Analysis of metagenome assemblies

The assembly of individual sequence reads into contigs and scaffolds is a powerful method for linking functional attributes with specific phylotypes. Assembly yielded scaffolds ranging from 1 kb (small contigs) to nearly 126 kb (largest scaffold), and an average scaffold size of 2,330 bp across all six sites. Community structure plays a role in the degree of assembly and the ability to obtain large scaffolds; communities with larger proportions of metagenome sequence originating from fewer, more dominant organisms resulted in longer assemblies. Diversity metrics of PCR-based 16S rRNA sequences that were produced simultaneously from the same samples indicated that subsurface mat communities from MS_15 and FG_16 exhibited higher Simpson’s diversity values (reported as the reciprocal of the Simpson’s index, λ^−1^; Table A1 in Appendix). The greater degree of species “evenness” in MS_15 and FG_16 yielded considerably smaller assemblies, and only two scaffolds >10 kb were obtained from each of these two sites. Contrastingly, CP_7 exhibited the lowest Simpson’s λ^−1^, and the largest assemblies were obtained from this site, which contributed 42% of the large scaffolds (>10 kb) obtained across all six sites. Large assemblies were also obtained from the anoxygenic mats at BLVA (BLVA_5, _20), and these samples had similarly low values for Simpson’s λ^−1^.

### Nucleotide word-frequency analysis of dominant populations

**Figure 3:**
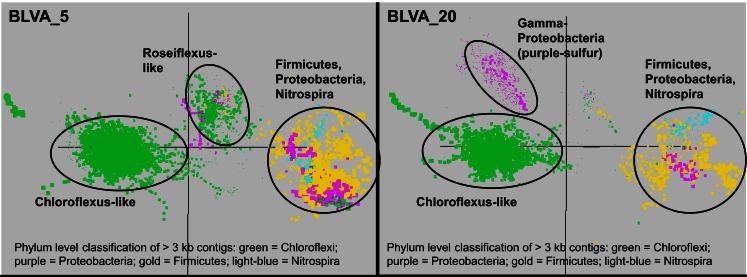
**Principal components analysis of oligonucleotide frequencies of assembled sequence from *Bath Lake Vista Annex***. BLVA_20 was sampled 8 months after BLVA_5 to capture a bloom of purple-sulfur bacteria shown in prior work to be related to *Thermochromatium tepidum* (Castenholz, 1977; Ward et al., 1989). Both sites contained scaffolds from dominant populations of *Chloroflexus* spp., Firmicutes, Nitrospira, and additional proteobacteria, but only BLVA_20 contained numerous scaffolds corresponding to the population of purple-sulfur bacteria (Gamma-proteobacteria, family *Chromatiaceae*, average G + C ∼64%) that is notably absent in BLVA_5.

**Figure A3:**
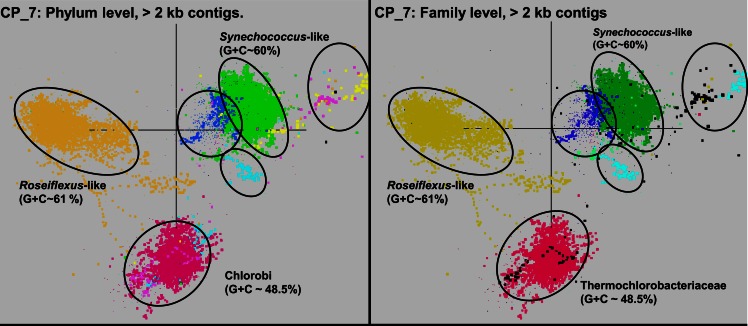
**Nucleotide word-frequency PCA of assembled sequence from Chocolate Pots (CP_7)**. This community contains predominant phylotypes of *Roseiflexus*-, *Synechococcus*-, “*Ca*. T. aerophilum”- (Chlorobi), and *Leptospirillum*-like populations as well as minor contributions from the Firmicutes, Proteobacteria, and Bacteroidetes [green = Cyanobacteria (*Synechococcus* spp.); gold = Chloroflexi (*Roseiflexus* spp.); maroon = Chlorobi/Thermochlorobacteriaceae/“*Ca*. T. aerophilum”, dark-blue = Spirochetes/*Leptospiraceae*; light-blue = Bacteroidetes/*Flexibacteraceae*; yellow = Firmicutes; light-purple = Proteobacteria].

**Figure A4:**
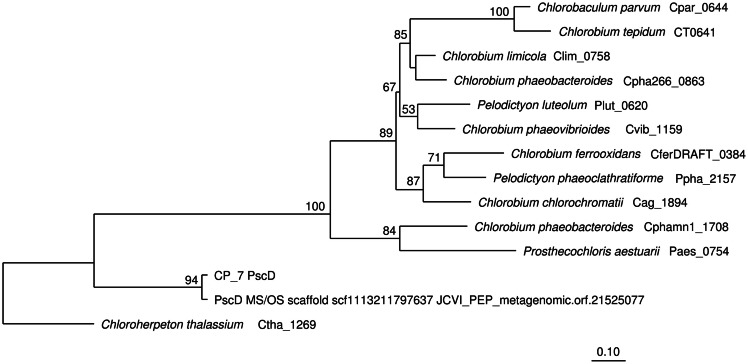
**Maximum-likelihood phylogenetic tree of Chlorobi PscD sequences from metagenomes and sequenced genomes**. The tree was produced using the program PhyML from PscD alignments containing 172 positions, with amino acid comparisons corrected using the Jones-Taylor-Thornton model (Jones et al., 1992). Bootstrap support for ≥50% of 1000 replicate trees are shown at nodes.

Sequence assemblies were examined using principal components analysis (PCA) of nucleotide word frequencies (NWF) (Teeling et al., 2004) in conjunction with a taxonomic classification algorithm of average scaffold identity (APIS; Badger et al., 2006). For example, NWF PCA plots of the sulfidic system at BLVA sampled 8 months apart revealed major differences in community composition associated with a visible bloom of purple-sulfur bacteria in BLVA_20 (Figures 1 and 3). The major change in community composition between the two samples was the *Thermochromatium*-like population in BLVA_20, which corresponded with a decrease in *Roseiflexus*-like sequences (Figure 3). Both BLVA samples revealed a dominant *Chloroflexus*-like population that corresponded to the G + C peak at 55% (Figure 2). Similar NWF PCA analyses of assemblies from CP_7 revealed three predominant community members related to *Roseiflexus*, *Synechococcus*, and “*Candidatus* Thermochlorobacter aerophilum”-like organisms (“*Ca*. T. aerophilum” represents a novel clade in the order Chlorobiales; Liu et al., 2012). Several other organisms were present in lower abundance and were distantly related to members of the Firmicutes, Bacteroidetes, and Spirochetes (Figure A3 in Appendix). The large Chlorobi-like assemblies obtained from CP_7 were phylogenetically related (average NT ID = 91%) to “*Ca*. T. aerophilum” assemblies obtained from *Mushroom* and *Octopus Springs* metagenomes (Klatt et al., 2011; Liu et al., 2012). Translated PscD sequences from this newly described lineage of uncultivated Chlorobi are clearly distinct from other previously described phototrophic Chlorobi (PscD sequences from the CP_7 and *Mushroom* populations have 95% amino acid identity (AA ID) (Figure A4 in Appendix).

**Figure 4:**
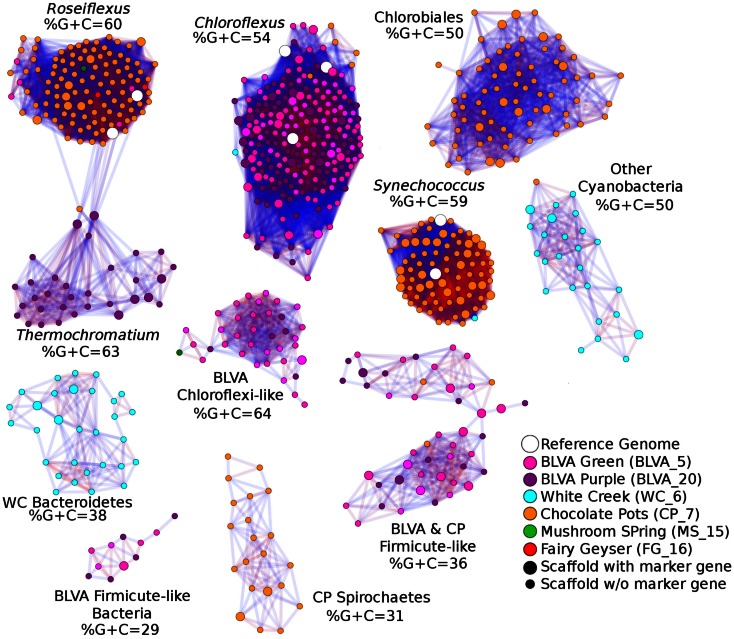
**Scaffold oligonucleotide frequency similarity network**. Oligonucleotide (tri-, tetra-, penta-, and hexa-nucleotide) counts were normalized to scaffold length and subjected to k-means clustering (*k* = 8, 100 trials). The scaffolds that group together in ≥90% trials are shown, with lines connecting scaffolds ranging from blue (90%) to red (100%). The sample origins of scaffolds shown here are indicated by site color (see legend) where open circles correspond to reference genomes; scaffolds containing phylogenetic or functional marker genes are indicated by larger nodes.

**Figure A5:**
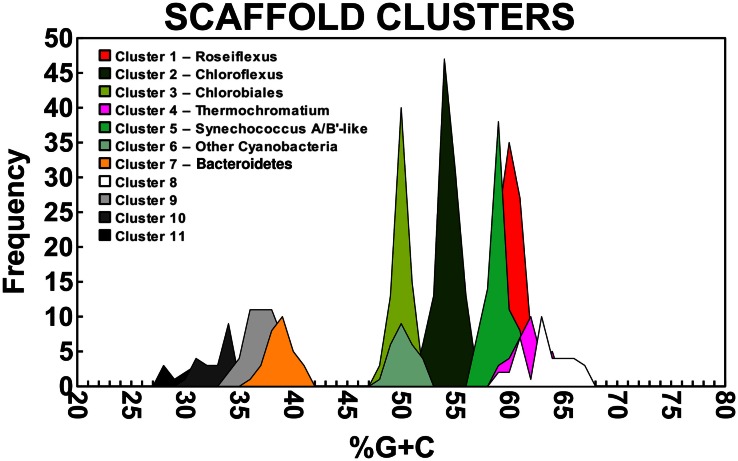
**G + C Composition of Scaffold Clusters**. Scaffold clusters greater than 10 kbp were demarcated using oligonucleotide frequencies as depicted in Figure 4.

A Monte-Carlo approach was also used to compare normalized oligonucleotide frequencies across the six phototrophic sites, which clustered the scaffolds of highly related organisms (e.g., genus/species level). A minimum scaffold length of 10 kbp was used to focus the analysis on dominant assemblies; consequently, smaller scaffolds from subsurface mat communities (MS_15 and FG_16) were not well represented in this analysis. Twelve scaffold clusters (consensus k-means groupings) were observed across sites (Figure 4; Table 2), and each of these populations corresponded with dominant community members identified using G + C content (%) and BLASTP assignments (Figure 2; Figure A5 in Appendix). Clustering by oligonucleotide frequency afforded greater discrimination among populations that exhibited similar G + C content. For example, *Roseiflexus*-like organisms have similar G + C content (61%) to *Synechococcus* sp. strains A and B′ (Figure 2), yet these different genera are clearly separated based on differences in sequence character using oligonucleotide clustering analysis (Figure 4).

**Table 2:** **Properties of scaffold clusters obtained from metagenome assemblies as demarcated with oligonucleotide composition and confirmed using phylogenetic analyses**.

Scaffold cluster	Taxonomic affiliation	Sites	No. of scaffolds	Median size (kbase)	G+C (%) ± SD	Total sequence (Mbase)	Depth of coverage (x)
1	*Roseiflexus* spp.	BLVA_5, CP_7, MS_15, FG_16	112	12.5	60.0 ± 1.2	1.55	2.6 ± 0.4
2	*Chloroflexus* spp.	BLVA_5, BLVA_20, WC_6, CP_7	211	13.5	54.3 ± 1.2	3.21	2.9 ± 0.7
3	*Ca*. Thermochlorobacter spp.	CP_7	73	14.8	49.5 ± 0.8	1.13	2.7 ± 0.5
4	*Thermochromatium* spp.	BLVA_20	29	12.5	63.0 ± 1.3	0.37	2.1 ± 0.4
5	*Synechococcus* spp.	WC_6, CP_7	78	26.2	58.7 ± 1.1	2.59	4.0 ± 0.7
6	Cyanobacteria	WC_6, CP_7	26	11.7	49.8 ± 1.2	0.32	2.4 ± 0.5
7	Bacteroidetes	WC_6	30	11.1	37.7 ± 0.9	0.37	2.4 ± 0.4
8	Chloroflexi-like	BLVA_5, MS_15, BLVA_20	37	10.6	63.9 ± 2.3	0.44	2.5 ± 0.5
9	Firmicute-like	BLVA_5, CP_7, BLVA_20	47	14.2	36.0 ± 1.5	0.79	2.7 ± 0.4
10	Firmicute-like	BVLA_5, BLVA_20	11	12.7	29.0 ± 1.4	0.16	2.6 ± 0.6
11	Spirochaetes	CP_7	21	11.8	30.5 ± 1.4	0.25	2.3 ± 0.4

*Scaffold clusters 7–11 represent novel bacteria that are not well represented in public databases, and are currently defined at the phylum level*.

**Figure A6:**
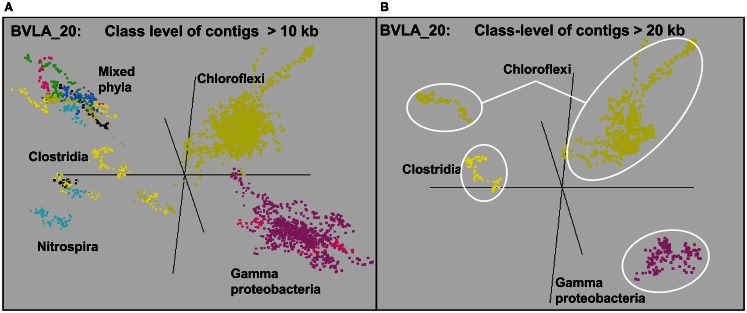
**Predominant metagenome sequence assemblies [(A) >10 kb contigs and (B) >20 kb contigs only] from Bath Lake Vista Annex (BLVA_20) sampled during a bloom of purple-sulfur bacteria (Gamma-proteobacteria)**. Major clusters are identified at the class-level (purple = Gamma-proteobacteria; gold = class Chloroflexi; yellow = Clostridia, light-blue = Nitrospira; green = Acidobacteria; maroon = Betaproteobacteria; dark-blue = Deltaproteobacteria).

A sequence cluster corresponding to *Thermochromatium* spp. (Gamma-proteobacteria) contained sequences solely from BLVA_20, which is consistent with visual evidence of this population at the time of sampling (Figure 1), as well as further NWF PCA analysis using contigs >20 kb (Figure A6 in Appendix). Other major sequence clusters identified included the *“Ca*. T. aerophilum”-like population from CP_7 (discussed above). Although relatives of the Bacteroidetes were found to occupy all sites, larger assemblies of several of these community members were obtained from WC_6. Three scaffold clusters with comparatively low G + C content (<40%) were observed, but neither AMPHORA (based on phylogenetic analysis) nor MEGAN (“blastx” alignments) could classify the sequences in these groups. This suggested that they originated from organisms that are currently poorly represented in public databases.

### Use of single-copy genes to demarcate dominant populations

**Figure 5:**
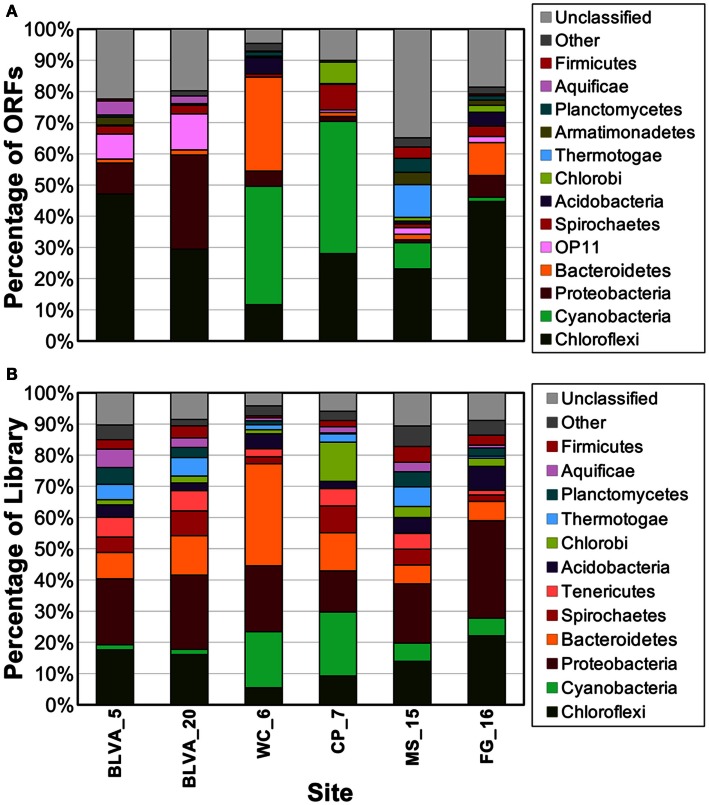
**Phylogenetic summary of marker genes from metagenome sequences compared to 16S rRNA gene sequences**. Phylogenetic marker genes in the metagenome sequences were **(A)** assigned and classified using AMPHORA, and compared to **(B)** 16S rRNA sequences from ribosomal panels (*n* ∼ 300 per site) classified at the phylum-level against the RDP at a confidence threshold of 80%.

Phylogenetically informative single-copy genes were identified among the metagenome assemblies using AMPHORA (Wu and Eisen, 2008), and provided yet another method for evaluating the predominant taxa represented in the six metagenomes. The distribution of dominant phylotypes predicted using AMPHORA (Figure 5A) was similar to that observed using the combined “blastx” and G + C (%) analyses of individual sequences (Figure 2), as well as to the taxonomic distribution of PCR-based 16S rRNA gene libraries from these same sites (Figure 5B). Moreover, the distribution of predominant populations (e.g., Chloroflexi, Cyanobacteria, Chlorobi, Proteobacteria) across sites was consistent with detailed analysis of major oligonucleotide clusters (e.g., Figures 3 and 4). All approaches showed that members of the Chloroflexi were ubiquitous across all sites. The relative contribution of *Chloroflexus* versus *Roseiflexus*-like organisms varied across different sites, and all sites contained novel organisms from undescribed lineages within the Chloroflexi (discussed in greater detail below). Other phototrophs detected in these sites included populations of Alpha-proteobacteria (Family *Hyphomicrobiaceae*) in FG_16, “*Ca*. C. thermophilum” (phylum Acidobacteria) (Bryant et al., 2007) in WC_6, and “*Ca*. T. aerophilum”-like organisms (order Chlorobiales) in MS_15, FG_16 and especially CP_7 (Figure 5B). The MS_15 community contained a *Thermotoga*-like population as well as several low G + C organisms that have not yet been characterized. Although the subsurface mat community from FG_16 contained a novel high G + C proteobacterial population not seen in the other sites (Figure 2), these sequences could not be linked unambiguously to the *Hyphomicrobiaceae* 16S rRNA sequences described above, due to inadequate sequence coverage of this population and the lack of a good reference genome that would undoubtedly have assisted in sequence identification.

**Figure 6:**
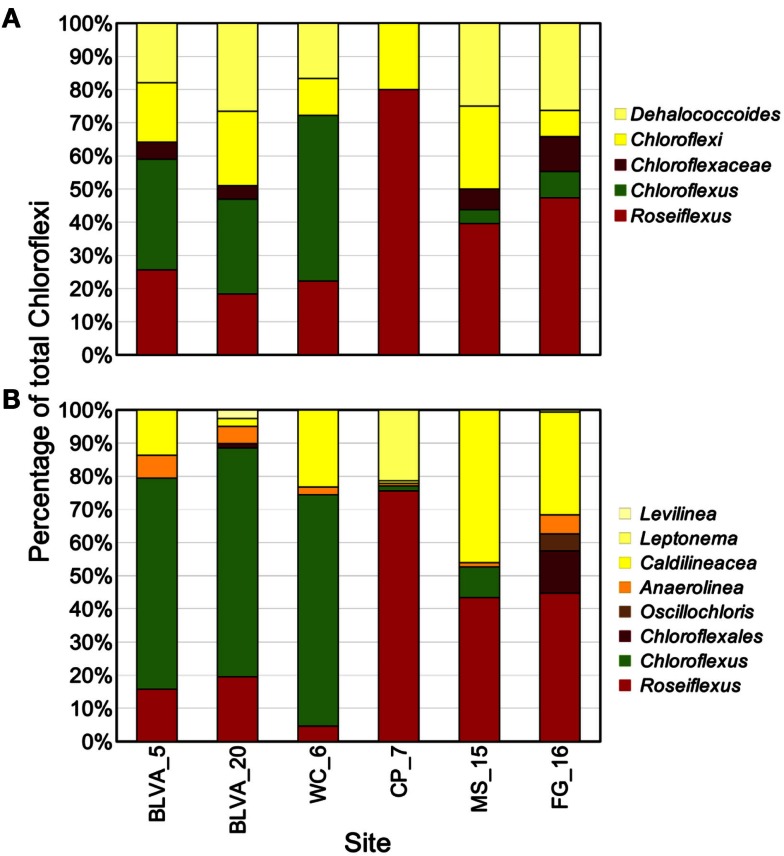
**Comparison of Chloroflexi phylogenetic marker genes from metagenomes and Chloroflexi 16S rRNA clones**. Phylogenetic marker genes within the metagenome sequences assigned to the phylum Chloroflexi using AMPHORA **(A)** compared to the identity (confidence threshold of 80%) of Chloroflexi-like 16S rRNA genes **(B)** observed in the ribosomal clone library (*n* ∼ 300 per site). Taxonomic groups of Chloroflexi: red = *Roseiflexus* spp., green = *Chloroflexus* spp., brown shades = other taxa within the order Chloroflexales, and yellow shades = other taxa within phylum Chloroflexi.

**Figure A7:**
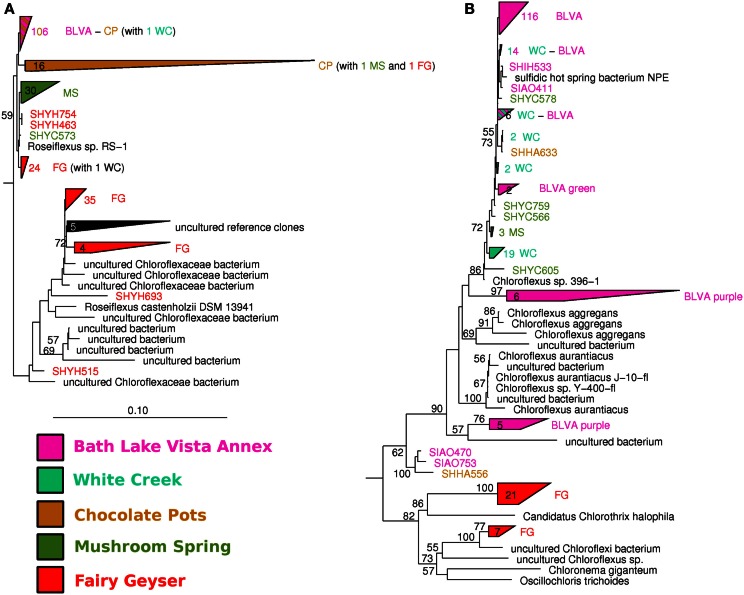
**Neighbor-joining phylogenetic tree of Chloroflexi 16S rRNA sequences from all clone libraries**. **(A)** Sub-branch of tree corresponding to *Roseiflexus* spp. **(B)** Sub-branch of tree corresponding to FAPs related to *Chloroflexus* spp. and other organisms capable of producing bacteriochlorophyll *c*. Sequences are color coded according to spring origin, and numbers adjacent to polygons indicate the number of clones in each clade. Bootstrap support for ≥50% of 1000 replicate trees are shown at nodes.

The distribution of phylogenetically unique Chloroflexi-like 16S rRNA gene sequences across sites was compared to the abundance of Chloroflexi marker genes in the metagenome assemblies identified using AMPHORA (Figure 6). The majority of Chloroflexi-like 16S rRNA sequences were most similar to either *Chloroflexus* or *Roseiflexus* spp.; however, many sequences fell outside of the family Chloroflexaceae and grouped with other members of the Chloroflexi that are not known to exhibit phototrophy (Figure 6). Additionally, *Roseiflexus*-like populations from MS_15, CP_7, and FG_16 and *Chloroflexus*-like populations from BLVA and WC_6 each formed monophyletic groups that excluded sequences from all other springs (Figure A7 in Appendix). Other spring-specific clades were observed for sequences from FG_16 within the class *Anaerolineae*, a group of Chloroflexi that was very recently shown to contain phototrophic members (Klatt et al., 2011). The presence of these 16S rRNA gene sequences, combined with observed Chloroflexi-like photosynthesis genes associated with these populations, suggests that these undescribed Chloroflexi may also contribute to phototrophy in these mat communities.

### Functional analysis of predominant sequence assemblies

#### Carbon fixation

The gene content of major scaffold clusters provides a basis for inferring the possible metabolic functions of dominant populations present in these communities (Table 3). For example, genes encoding key enzymes involved in the 3-hydroxypropionate (3-HP) pathway of inorganic carbon fixation were present in the metagenomes from all six sites, and were associated with the predominant *Chloroflexus* and *Roseiflexus-*like populations present in these habitats. Genes coding for subunits of ribulose 1,5-bisphosphate carboxylase-oxygenase (RuBisCO), a key enzyme in the reductive pentose phosphate pathway (i.e., Calvin-Benson-Bassham cycle) were observed only in cyanobacterial (WC_6 and CP_7) or proteobacterial sequences (alpha-proteobacteria and *Thermochromatium* spp. in FG_16 and BLVA_20, respectively). No CO_2_ fixation genes were associated with the sequences derived from the “*Ca*. T. aerophilum”-like populations from CP_7, despite the fact that other cultivated members of this phylum are capable of fixing CO_2_ via the reductive tricarboxylic acid (rTCA) cycle. The average coverage of “*Ca*. T. aerophilum”-assemblies (∼3×) may not be sufficient to conclude that these Chlorobi definitively lack the capacity to fix inorganic carbon, however, metatranscriptomic studies with much deeper coverage also failed to identify key genes (i.e., ATP-citrate lyase) of the rTCA cycle in these populations at *Mushroom Spring* (Liu et al., 2012). This organism is a member of a novel, family level lineage of the Chlorobi, which are predicted to be aerobic photoheterotrophs that cannot oxidize sulfur compounds, cannot fix N_2_, and do not fix CO_2_ autotrophically (Liu et al., 2012).

**Table 3:** **Phylogenetic distribution of autotrophic, phototrophic, and sulfur cycling genes in metagenomes**.

Phylogenetic Group	BLVA Green (BVLA_5)	BLVA Purple (BVLA_20)	White Creek (WC_6)	Chocolate Pots (CP_7)	Mushroom Spring (MS_15)	Fairy Geyser (FG_16)
**CARBON FIXATION PATHWAYS**						
*Roseiflexus* spp.	**0.60**	**0.80**	0.20	**0.90**	**0.60**	**0.80**
*Chloroflexus* spp.	**1.00**	**1.00**	**0.80**			
Other Chloroflexi				0.20		**0.50**
Cyanobacteria			0.20	**0.60**		
*Thermochromatium* spp.		**0.67**				
Alpha-proteobacteria						**0.67**
**(BACTERIO)CHLOROPHYLL BIOSYNTHESIS**						
*Roseiflexus* spp.	**0.76**	**0.57**	0.14	**0.86**	**0.57**	**0.76**
*Chloroflexus* spp.	**1.00**	**0.90**	**0.76**		0.19	0.05
Other Chloroflexi			0.14	0.14	0.14	0.43
*Thermochromatium* spp.		0.13				
Cyanobacteria			0.45	**1.36**	0.27	0.09
*Ca*. Thermochlorobacter spp.			0.08	**0.83**	0.08	0.25
*Ca*. Chloracidobacterium spp.			0.23		0.05	
Alpha-proteobacteria						0.25
**PHOTOSYSTEM REACTION CENTERS**						
*Roseiflexus* spp.	**0.50**	0.25		**0.50**		**0.50**
*Chloroflexus* spp.	0.40	0.40	0.20			
Cyanobacteria			0.33	**0.97**	**0.52**	**1.00**
**SULFUR CYCLING**						
*Thermochromatium* spp.		**0.80**				

*Entries represent relative completeness of indicated pathways calculated as the fraction of a unique occurrence of a gene in a taxon divided by the total number of genes known to be involved in that function (values > 0.5 are in bold). Metagenome sequences were compared to known pathways in the genome sequences of Chloroflexus aurantiacus J-10-fl, Roseiflexus sp. strain RS-1, Thermochromatium spp., Allochromatium vinosum, Synechococcus sp. strain A, “Candidatus Chloracidobacterium thermophilum”, Chloroherpeton thalassium, and the alpha-proteobacterium, Rhodopseudomonas palustris TIE-1*.

#### Chlorophototrophy

Genes involved in (bacterio)chlorophyll biosynthesis and the production of photosynthetic reaction centers (here termed chlorophototrophy genes) were present in scaffold clusters corresponding to *Roseiflexus*, *Chloroflexus*, *Thermochromatium*, and *Synechococcus* spp., as well as the “*Ca*. T. aerophilum”-like population in CP_7, and other Cyanobacteria, especially in WC_6 (Table 3). Consequently, the dominant phototrophs within each community exhibit genomic capability for chlorophototrophic metabolism. Examination of shorter (<10 kbp) scaffolds revealed additional genes involved in chlorophototrophy, and these were assigned to specific chlorophototrophic organisms such as “*Ca*. Chloracidobacterium spp.” present in WC_6, and uncultivated proteobacteria in the FG_16 subsurface mat community (Table 3). The high G + C% proteobacterial sequences from FG_16 averaged 74% identity (AA) to *Rhodopseudomonas palustris* and other alpha-proteobacterial genomes, and are likely contributed from the *Hyphomicrobiaceae* population in FG_16. Genes from Chloroflexi coding for chlorophototrophic functions, but too divergent to originate from either *Chloroflexus* or *Roseiflexus* spp. (i.e., only ∼70% AA ID), were present in all non-sulfidic sites, especially in FG_16 (Table 3). The Chloroflexi-like chlorophototrophy genes from FG_16 are phylogenetically distinct (<70% AA ID) from previously described metagenome sequences and all related sequences residing in public databases, indicating that novel uncultured phototrophic members of the Chloroflexi inhabit the mats at *Fairy Geyser*. Three deduced protein sequences from the subsurface layer in *Mushroom Spring* (MS_15) were highly similar (96–100% AA ID) to translated sequences of novel chlorophototrophy genes observed in recent “meta-omic” studies of the top-layers of this same mat type (Klatt et al., 2011; Liu et al., 2011); these observations linked these genes to a group within the Chloroflexi not previously known to contain chlorophototrophic organisms.

#### Iron oxidation

One goal of this study was to investigate the role of anoxygenic photosynthesis in sulfidic communities from *Bath Lake Vista Annex* and in iron mats at *Chocolate Pots*. Previous studies near the source of *Chocolate Pots* (and near CP_7) have shown that the oxidation of aqueous Fe(II) is abiotic, but mediated by the production of oxygen by cyanobacteria (Pierson et al., 1999; Trouwborst et al., 2007). However, voltammetric microelectrode studies revealed that Fe(II) persists in deeper layers of the mat, providing a potential niche for anoxygenic phototrophs that can use Fe(II) as an electron donor for photosynthesis (photoferrotrophy) (Trouwborst et al., 2007). Query genes for both sulfur and Fe(II) oxidation (Croal et al., 2007; Jiao and Newman, 2007; Frigaard and Dahl, 2009; Grimm et al., 2011; Bryant et al., 2012) were used to search for evidence of sulfide or Fe(II) oxidation in the community from CP_7. No genes with significant similarity to the photosynthetic iron oxidation (*pio*) operon of the purple non-sulfur *Rhodopseudomonas palustris* TIE-1 (Jiao and Newman, 2007) or the *fox* operon of the purple non-sulfur *Rhodobacter ferrooxidans* SW2 (Croal et al., 2007) were observed in CP_7, or any site described in this study with the exception of one sequence in FG_16, a site that contains below detectable levels of iron (Table 1). This result concurs with the low numbers of alpha-proteobacterial sequences in CP_7 (Table 3), and the lack of Fe(II) oxidation when similar mats were illuminated with near-infrared radiation to excite bacteriochlorophylls (Trouwborst et al., 2007). To date, no thermophilic representatives of purple and green photoferrotrophs have been discovered.

#### Sulfur oxidation

Genes known to encode proteins involved in sulfur oxidation (*dsr* complex) in some anoxygenic phototrophs (e.g., gammaproteobacterium *Allochromatium vinosum*, Dahl et al., 2005; Frigaard and Dahl, 2009; Gregersen et al., 2011) were identified in the *Thermochromatium*-like population from BLVA_20, and this is consistent with the high concentrations of DS (>100 μM) measured *in situ*. However, the dominant *Chloroflexus*-like populations observed in both *BLVA* samples do not contain *dsr* or *sox* genes known to be involved in the oxidation of reduced-sulfur compounds. This is consistent with the absence of these same genes in reference *Chloroflexus* and *Roseiflexus* spp. genomes (van der Meer et al., 2010; Tang et al., 2011). However, the *Chloroflexus* assemblies from BLVA_20 and *Roseiflexus* assemblies of CP_7 (as well as FAP reference genomes) contain *sqr* genes, which encode sulfide-quinone oxidoreductases and have been suggested to play a role in the oxidation of sulfide to elemental sulfur in multiple bacterial phyla (Griesbeck et al., 2002; Chan et al., 2009; Marcia et al., 2009). Consequently, it is possible that proteins encoded by *sqr* genes may enable FAPs to obtain electrons from reduced-sulfur compounds (Frigaard and Dahl, 2009; Gregersen et al., 2011; Bryant et al., 2012). In the current study, the presence of similar *Chloroflexus* as well as similar *Roseiflexus* populations across both sulfidic and non-sulfidic sites argues that utilization of sulfide as an electron source is not an obligate physiological trait across these genera.

#### Anaerobic metabolism

Sequence clusters corresponding to undescribed organisms from the Bacteroidetes show no evidence of chlorophototrophy, but rather contain genes suggestive of anaerobic metabolism(s). Protein-coding genes involved in the oxidation and/or fermentation of organic acids were noted in several sites. For example, acyl-CoA synthetases and lactate dehydrogenases were found in unidentified clusters from BLVA (G + C = 64%) and CP_7 (G + C = 31%) and a mixed cluster containing sequences from BLVA and CP (G + C = 36%). Subunits of a pyruvate ferredoxin: oxidoreductase (PFOR) were found in both unidentified BLVA clusters. Although important in every mat type, insufficient coverage of the less-dominant anaerobic populations present in chlorophototrophic mats precludes a thorough analysis of their metabolic potential.

### Comparative analysis of protein families

**Figure 7:**
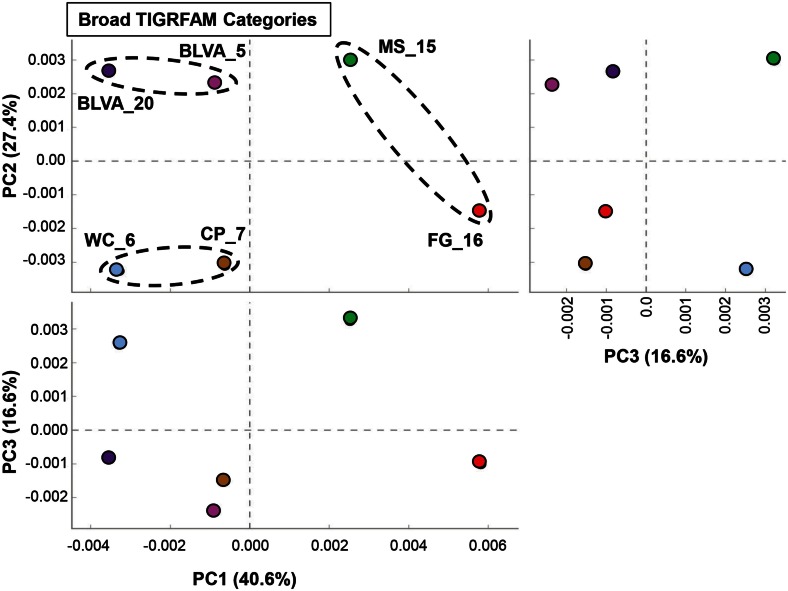
**Principal components analysis (PCA) of relative gene abundances (TIGRFAMs) across six phototrophic sites**. Principal components (PC1, PC2, PC3) obtained across all TIGRFAMs grouped into functional categories (also see Figure A8 in Appendix for hierarchal cluster analysis). Site-pairs are circled based on separation achieved with PC1 and/or PC2 (BLVA_5 = fuschia, BLVA_20 = purple, WC_6 = light-blue, CP_7 = gold-brown, MS_15 = green, FG_16 = red).

A complete functional analysis was performed (using multivariate statistical analysis) by assigning TIGRFAM protein families to predicted proteins within all metagenome assemblies. Differences in gene contents among the six chlorophototrophic mats should be indicative of changes in community structure and the corresponding functional attributes of dominant community members. PCA was used to examine the relative differences among sites based on all TIGRFAM categories (Figure 7). Factor 1 (PC1, accounting for ∼41% of the relative functional variation across sites) separates subsurface from surface mat communities, while PC2 (∼27% of variation) separates the sites according to different levels of oxygen (or sulfide) and the presence of oxygenic phototrophs. Factor 3 (PC3, ∼17% of variation) emphasizes functional similarities between MS_15 and WC_6 that are difficult to separate based only an examination of the abundance of different phylotypes across these sites (e.g., Figure 2). For example, although both sites contained cyanobacteria (e.g., low sulfide), MS_15 contained more sequences related to *Roseiflexus* spp., while WC_6 contained numerous *Chloroflexus-*like sequences. These populations may be organotrophic in this environment and not dependent on sulfide or elemental sulfur (Table 1; Figure 6).

**Figure 8:**
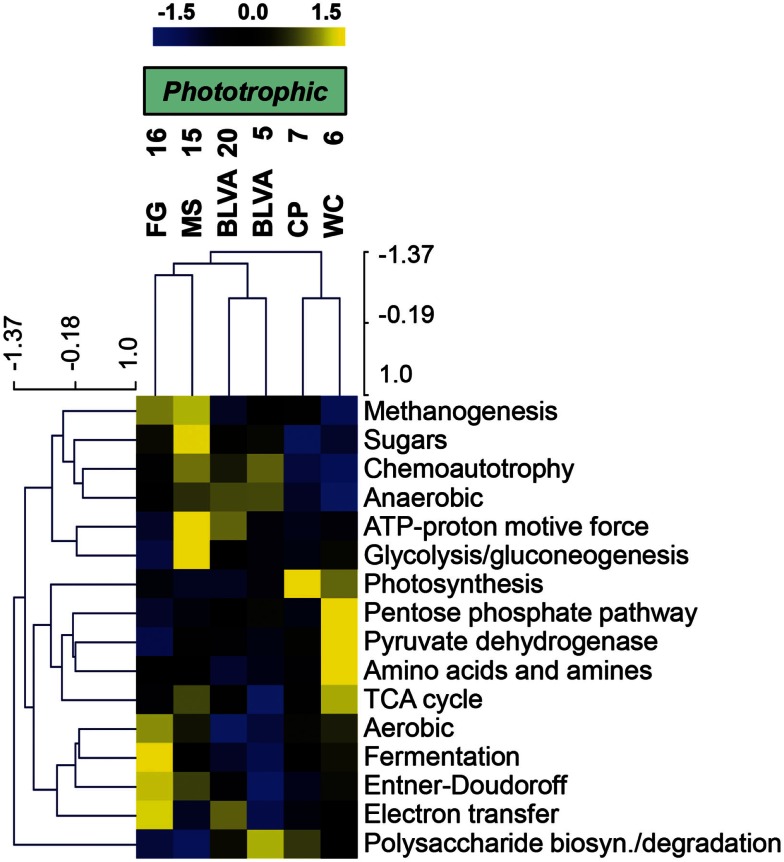
**Hierarchical cluster analysis of relative abundances of genes in TIGRFAMs associated with “Energy Metabolism” classified by functional category**. Data was standardized by functional category before clustering to avoid biasing analysis by a few categories with high gene abundance. Pearson correlation was used as the distance measure for average linkage agglomerative clustering.

**Figure A8:**
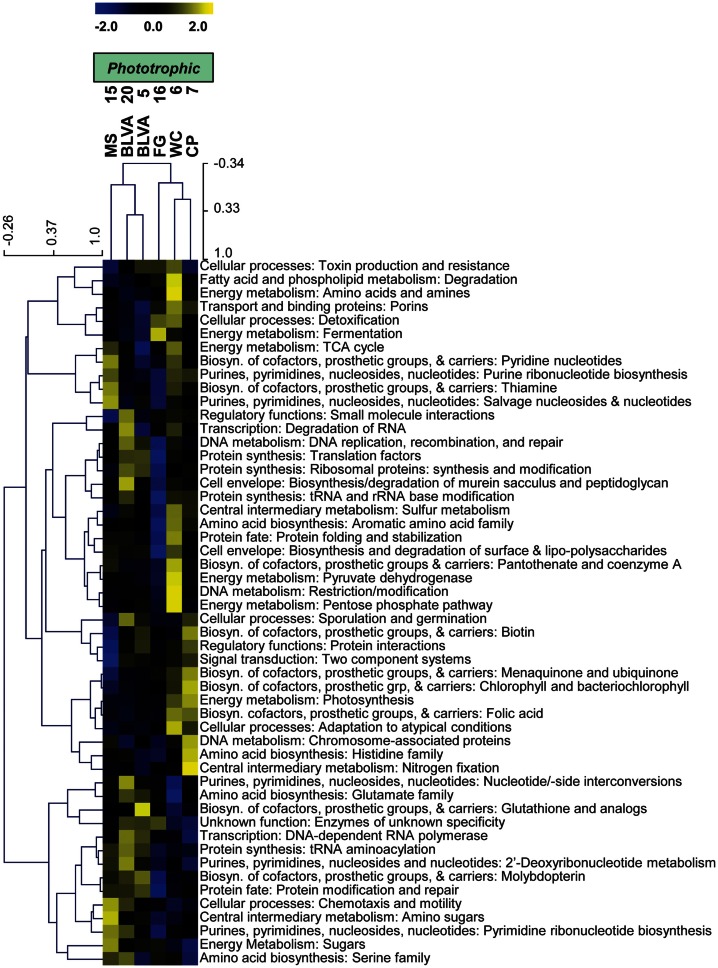
**Hierarchical cluster analysis of relative abundances of genes in all TIGRFAMs grouped into functional categories**. Data were standardized by functional category before clustering to avoid biasing analysis by a few categories with high gene abundance. Pearson correlation was used as the distance measure for average linkage agglomerative clustering.

Specific TIGRFAM categories responsible for differences across sites were also evaluated using hierarchical cluster analysis. Two approaches were evaluated using either a smaller set of TIGRFAM categories related to “energy metabolism” (Figure 8) or all TIGRFAM families (Figure A8 in Appendix). In each case, communities (sites) clustered as expected based on replication of specific variables such as sulfide/oxygen, temperature, and mat sample depth (Inskeep et al., 2013). The relative abundance of TIGRFAMs associated with “energy metabolism” was evaluated and included genes related to sugar degradation, glycolysis/gluconeogenesis, pentose phosphate pathway, fermentative processes, electron transport, and chemolithoautotrophy (Figure 8). Site clustering using these TIGRFAMs confirmed greater metabolic potential for processes such as aerobic metabolism and oxygenic photosynthesis in CP_7 and WC_6, samples that contained the most cyanobacteria (e.g., *Synechococcus*, *Fischerella*). Conversely, the subsurface mat communities (FG_16 and MS_15) exhibited a greater abundance of genes related to the Entner-Doudoroff pathway and fermentative processes, which are expected to be more important in subsurface environments occurring just below the predominant cyanobacterial populations (See Materials and Methods). Relative abundance within the TIGRFAM category “aerobic metabolism” revealed greater numbers of these genes in sites that contained significant levels of dissolved oxygen (i.e., no DS) compared to sulfidic sites (BLVA_5, 20). Moreover, TIGRFAMs associated with “anaerobic metabolism” as well as “chemoautotrophy” were higher in the sulfidic sites (BLVA sites 5 and 20) (Figure 8), although some of these TIGRFAMs are also present in subsurface mat communities. As should be clear, specific inferences on the basis of a TIGRFAM assignment must be followed with further analysis of the specific gene or set of genes responsible for the abundance estimates within a category.

Hierarchical cluster analysis across all TIGRFAMs grouped into 52 functional categories showed generally similar results regarding site clustering, but the number of TIGRFAM categories used in the analysis precludes a full description of all protein families (Figure A8 in Appendix). Based on clear differences in the phylotypes observed in sulfidic (hypoxic) vs. oxic samples, the TIGRFAM abundance profiles from BLVA (sites 5 and 20), and those from CP_7 and WC_6 formed separate clusters as expected. However, relative TIGRFAM abundance profiles of the subsurface mat communities (FG_16 and MS_15) did not form a separate cluster, as these sites simply do not exhibit greater similarity to one another compared to similarity among all sites (e.g., organisms similar to *Roseiflexus* spp. are present in all sites). Despite similarities in physical context, the two subsurface communities (MS_15, FG_16) revealed different functional signatures consistent with substantial differences in community composition described above (Figure 2), and that are likely due to differences in geochemistry and temperature between the two samples (FG_16 is ∼15°C cooler than MS_15 and exhibits higher pH values, above pH 9). Consequently, the functional profiles across all TIGRFAM groupings are consistent with, and provide further support for, the differences in community structure between MS_15 and FG_16 (Figure A8 in Appendix).

## Discussion

The six sites investigated in this study are representative of three general types of geothermal springs in Yellowstone National Park that support bacterial chlorophototrophic communities and include (i) alkaline-siliceous chloride springs (pH 7.5–9; e.g., WC_6, MS_15, and FG_16), (ii) sulfidic-carbonate springs (pH 6–7; e.g., BLVA_5 and BLVA_20), and (iii) mildly acidic (pH 6) non-sulfidic springs containing high aqueous Fe(II) (e.g., CP_7) (Rowe et al., 1973; McClesky et al., 2005). The major physical and geochemical constraints that have been postulated to control the distribution of phototrophs (and photosynthesis) in these thermal springs are pH, temperature, sulfide concentration, and gradients in light and/or other chemicals existing as a function of mat depth (Brock, 1967, 1978; Cox et al., 2011; Boyd et al., 2012). The upper temperature limit of cyanobacterial photosynthesis is known to occur at ∼74°C (Brock, 1973), and the grazing of these microbial mats by eukaryotic organisms typically only occurs at temperatures below 50°C. Most springs that support bacterial chlorophototrophic mats occur at pH > 5, with rare exceptions such as the acid-tolerant, purple non-sulfur phototrophs related to *Rhodopila* sp. observed in *Nymph Lake* (YNP) and in small sulfidic, acidic (pH 3.5–4.5) springs near the *Gibbon River* (Pfennig, 1974; Madigan et al., 2005). The bulk aqueous pH at CP_7 is near the lower limit observed for thermophilic cyanobacteria (Brock, 1973), and microelectrode measurements of the CP_7 mat revealed that it was constantly flushed by vent water with a pH ∼ 6 (Trouwborst et al., 2007). Even at pH 6, CP_7 supports an active community of cyanobacteria that are similar to *Synechococcus* sp. B′-like populations observed in *Mushroom* and *Octopus Spring* (pH > 8) phototrophic mats (Figure A1 in Appendix).

### Distribution of anoxygenic phototrophs

Anoxygenic chlorophototrophs are known to colonize sulfidic springs of YNP (van Niel and Thayer, 1930; Castenholz, 1969, 1977; Madigan, 1984; Giovannoni et al., 1987), and this was confirmed in samples from BLVA in which concentrations of DS exceeded 100 μM. However, the only population with genes supporting a complete, well-studied sulfide-oxidization pathway (Dahl et al., 2005) was the *Thermochromatium*-like organisms present in BLVA_20. The other prominent anoxygenic chlorophototrophs included populations of *Chloroflexus* and *Roseiflexus*-like spp. (identified across all sites). The abundance of chlorophototrophic Chloroflexi across sites is reflective of their previously established physiological diversity, including photoheterotrophy with organic acids such as acetate and propionate, photoautotrophy, photomixotrophy, and oxic and anoxic chemoorganotrophy (Madigan et al., 1974; Pierson and Castenholz, 1974; Giovannoni et al., 1987; Hanada et al., 2002; van der Meer et al., 2003, 2010; Zarzycki and Fuchs, 2011). While these organisms are generally photoheterotrophic, their metabolic flexibility contributes in part to their ability to colonize a broad spectrum of slightly acidic to neutral pH environments at 50–70°C (Castenholz and Pierson, 1995). Highly similar (>98% average NT ID) *Roseiflexus*-like organisms were abundant in all sites, independent of bulk sulfide concentration. Moreover, *Chloroflexus*-like populations were found in both sulfidic (BLVA) and oxic systems (WC_6). The presence of *Roseiflexus* spp. sequences in BLVA_5 and _20 and the larger proportion of *Chloroflexus* spp. in WC_6 compared to *Roseiflexus* spp. was unexpected, as it has been shown that *Chloroflexus* spp. tolerate higher levels of sulfide in culture (Madigan et al., 1974; Giovannoni et al., 1987; van der Meer et al., 2010). These results suggest that sulfide concentration is not a deterministic variable explaining niche partitioning between *Chloroflexus* spp. and *Roseiflexus* spp. This inconsistency with expected distribution patterns implies that factors other than sulfide and/or oxygen are important in controlling the relative abundance of *Chloroflexus* and *Roseiflexus* spp. in YNP phototrophic mat environments. Finally, sequences assigned to “*Ca*. C. thermophilum” (phylum Acidobacteria) (Bryant et al., 2007) were most abundant in the oxic communities of WC_6 and MS_15 (∼8 and 3% of sequences, respectively). Although small numbers of sequences (<1%) assigned to this organism (BLASTN, >50% NT ID) were observed in other sites, genes encoding enzymes of (B)Chl biosynthesis and belonging to “*Ca*. C. thermophilum” were only found in WC_6 and MS_15 (Table 3).

The observed differences in functional gene content between the two subsurface mat communities (MS_15 and FG_16) were of further interest, in part due to the presence of different poorly understood organisms in both sites. “Red-layer” communities (FG_16) have been shown to contain novel phototrophs (Boomer et al., 2000, 2002), whose pigments exhibit unusual *in vivo* absorption spectra (Boomer et al., 2000). Indeed, the FG_16 sample contained a high G + C (∼68–70%) alpha-proteobacterial population not observed in any other site (Figure 2). The 16S rRNA sequences from FG_16 indicated the presence of an alphaproteobacterium (family *Hyphomicrobiaceae*), some members of which are known to produce BChl *b* (Hiraishi, 1997). BChl *b* pigments were detected in solvent-based extractions from *Fairy Geyser* mat samples (M. Pagel and D. A. Bryant, unpublished data) and suggest that the phototrophs producing these pigments may exhibit light-harvesting properties that differ from those of other chlorophototroph populations in the mats.

Differences in community composition between the two subsurface mat communities may be driven by differences in temperature (60 vs. 36–40°C in MS_15 and FG_16, respectively). However, the MS_15 subsurface community was also distinct from surface (top 1–2 mm) communities sampled from the same mats at the same temperature (Klatt et al., 2011). For example, the abundance of *Thermotoga* spp. in the subsurface communities may be driven primarily by lower oxygen levels shown to exist 2 mm below the mat surface (Jensen et al., 2011) and is consistent with their physiology as microaerophilic heterotrophs (van Ooteghem et al., 2004). Anaerobic fermentation by *Thermotoga* spp. could constitute a major source of H_2_ that could enable photomixotrophic metabolism by *Chloroflexus* and *Roseiflexus* spp. (Klatt et al., 2013). Moreover, compared to the phototrophic surface layers of these mats, MS_15 subsurface communities contained fewer *Synechococcus* spp., greater *Roseiflexus* spp., and greater numbers of likely anaerobic or fermentative organisms within the Bacteroidetes and Thermodesulfobacteria.

### Trophic interactions

Trophic interactions between FAPs and cyanobacteria have been studied in phototrophic geothermal mats, and it has been shown that photoheterotrophs (FAPs) utilize organic acids produced by autotrophic cyanobacteria (Anderson et al., 1987; Nold and Ward, 1996; van der Meer et al., 2005). Moreover, it has been proposed that *Thermochromatium* spp. (purple-sulfur bacteria) are primary producers in sulfidic springs and cross-feed low-molecular weight organic acids to FAPs (Madigan et al., 1989, 2005). This is analogous to the cyanobacterial primary production and trophic interactions documented to occur in *Octopus Spring* and *Mushroom Spring* (van der Meer et al., 2005). However, this hypothesis is not supported by the relatively heavy carbon isotope composition of Chloroflexaceae-specific lipid biomarkers in sulfidic springs (δ^13^C = −8.9 to −18.5 ‰, van der Meer et al., 2003). These isotopic compositions have been interpreted to be too heavy to originate from compounds cross-fed from *Thermochromatium* spp., which use the Calvin-Benson-Bassham cycle for carbon dioxide fixation (δ^13^C = −20 to −35 ‰). The lipid signatures are more readily explained by direct carbon dioxide fixation by *Chloroflexus* and *Roseiflexus* spp. via the 3-HP pathway (Holo and Sirevåg, 1986; Strauss and Fuchs, 1993; van der Meer et al., 2000, 2010). Metagenome sequence assemblies obtained in the current study showed that these uncultivated *Chloroflexus* and *Roseiflexus* spp. contained all genes necessary for CO_2_ fixation via the 3-HP pathway (Table 3), and is consistent with earlier evidence at *BLVA* of short-term, sulfide-stimulated ^14^CO_2_ incorporation by FAPs (Giovannoni et al., 1987). Collectively, these observations support the hypothesis that all major chlorophototrophs contribute to primary productivity in sulfidic-carbonate springs (Table 3). It remains to be determined whether FAPs are more important contributors to primary productivity in these systems when purple-sulfur bacteria (i.e., *Thermochromatium*) and cyanobacteria are both absent (such as observed in BLVA_5).

This study highlights several of the major differences in community composition and structure, and potential function of chlorophototrophic microbial mats sampled from high-temperature systems (40–60°C) containing high sulfide, high Fe(II), or high dissolved oxygen. The distribution of chlorophototrophic organisms, as would be expected, is dependent on the presence or absence of high sulfide (cyanobacteria, purple-sulfur bacteria), and position within laminated mats (e.g., FAPs, Bacteroidetes, and Firmicutes). Temperature was not particularly well constrained as a consistent parameter for comparisons across the sites included in this study. However, the ubiquity of *Chloroflexus* and *Roseiflexus* spp. across all sites emphasizes their ability to tolerate large differences in not only temperature, but extremes between high and low levels of DS and/or oxygen. Assemblies of a novel Chlorobi population (“*Ca*. T. aerophilum”) from the high iron site at *Chocolate Pots* (CP_7) were similar to those obtained from *Mushroom Spring* and *Octopus Spring* (Liu et al., 2012). These populations deserve further study, especially considering their phylogenetic distance and different functional attributes compared to other currently described members of the Chlorobi. The dominant cyanobacteria observed across these sites (found exclusively in non-sulfidic systems) included *Synechococcus* spp. (CP_7, MS_15) and *Fischerella* (*Mastigocladus*) spp. (WC_6). Consequently, sulfide is a critical geochemical variable that selects against the presence of cyanobacteria and provides niche opportunities for other chlorophotoautotrophs. Other poorly represented organisms in the current study include bacteria from the phyla Firmicutes and Bacteroidetes, and although the assemblies for organisms within these phyla were not particularly large, a sufficient number of genes were found to infer that their role in these communities may involve fermentation and the degradation of complex carbon compounds. Additional sequence assembly and/or isolation of these populations, coupled with site-specific studies, are necessary to clarify the important carbon cycling functions that these populations conduct and the processes that drive interactions among primary producers and secondary consumers in chlorophototrophic mats.

## Materials and Methods

### Sample collection and geochemical analyses

Six different samples were obtained from five hot springs between August 2007 and May 2008 (Table 1; Table S2 in Inskeep et al., 2013) and immediately frozen in liquid N_2_. Phototrophic mats were sampled at different locations relative to the source of each respective spring, and two samples were obtained from subsurface mat layers [*Mushroom Spring* (MS_15) and *Fairy Geyser* (FG_16)]. The subsurface layers were obtained by careful removal of the top 2 mm green layer with a sterile scalpel and separation of a definitive under-layer in each mat type (e.g., Boomer et al., 2000, 2002; Nübel et al., 2002). Geochemical characterization was performed on bulk spring water at each sampling location after filtration (0.2 μm). Total dissolved ions were determined using inductively coupled plasma spectrometry and major anions determined using ion chromatography as described previously (Macur et al., 2004; Inskeep et al., 2005). Temperature, pH, total DS, total soluble Fe, and dissolved oxygen were determined immediately in the field. Dissolved gases (CO_2_, CH_4_, and H_2_) were determined using headspace gas chromatography of filtered field samples (Inskeep et al., 2005).

### DNA extraction and preparation

Environmental DNA was extracted as described in Inskeep et al. (2013). Briefly, 0.5–1 g of frozen mat samples were processed using separate parallel DNA extractions with an enzymatic method (Proteinase K (1 mg/ml) with Na-dodecyl sulfate (SDS) (0.3% w/v) for 0.5 h at 37°C) and a mechanical method (bead-beating with 2% w/v SDS and 15% v/v Tris-HCl-equilibrated phenol, shaken at 5.5 m/s for 30 s) for cell lysis. The resulting cell lystates were pooled and subsequent DNA extractions were performed with phenol:chloroform:isoamyl alcohol (25:24:1), and chloroform:isoamyl alcohol (24:1). This procedure removed DNA extraction bias that has been shown to occur when only mechanical or enzymatic protocols are used for cell lysis (Klatt et al., 2007, 2011). All samples were treated with RNAse I (Promega, Madison, WI, USA), and DNA was precipitated with ethanol and Na-acetate. Small-insert (3 kb) metagenome libraries were constructed as described in Inskeep et al. (2013). About 820 bp was sequenced at each end of the inserts in the library clones, which produced pairs of linked sequences (424,982 sequences) that represented a total dataset of ∼320.6 Mbp. Ribosomal (16S rRNA) gene sequence libraries were constructed by PCR amplification using universal primers targeting domains Archaea (4aF, TCCGGTTGATCCTGCCRG; 1391R, GACGGGCRGTGWGTRCA) and Bacteria (27F, AGAGTTTGATCCTGGCTCAG and 1391R). Amplicons were cloned using the TOPO TA Cloning Kit (Invitrogen, Carlsbad CA USA) and sequenced using Big Dye v3.1 chemistry (Applied Biosystems, Foster City, CA, USA).

### Pre-assembly metagenome sequence analyses

All metagenome sequences were used as queries in a “blastx” (Camacho et al., 2009) search against the NCBI nr database (accessed 22 March 2011) with default parameters. The results were parsed and visualized with the MEGAN software version 2.3.2 (Huson et al., 2007) with the default parameters (MinScore = 35.0, TopPercent = 10.0, MinSupport = 5) and taxonomic assignments of the top “blastx” matches were extracted. Comparative analysis was also completed using several relevant reference genomes available after this date (e.g., *Fischerella* sp. and “*Ca*. T. aerophilum”; Liu et al., 2012).

### Sequence assembly and annotation

Metagenomic scaffolds of overlapping end sequences were constructed separately for each of the six samples using the Celera assembler (Miller et al., 2008; Inskeep et al., 2013). This resulted in 206,469 scaffolds containing 183.2 Mbp (27–33 Mbp per site) of assembled sequence, or a 57% compression of the raw sequence data. The DOE-JGI annotation pipeline was used as an initial step for inferring functions for predicted ORFs on metagenome scaffolds, and included open reading frame (ORF) prediction, BLAST alignments, and hidden Markov model analysis (Mavromatis et al., 2009). Translated peptide sequences from predicted ORFs were analyzed with the AMPHORA package (Wu and Eisen, 2008), which identified homologs of 31 different genes (mostly predicted to encode ribosomal proteins or enzymes with housekeeping functions) that could be used as phylogenetic markers. Genes encoding particular functions were identified by BLASTP using reference sequences as queries, with the additional requirement that candidate sequences had a top BLASTP match to a sequence with the same annotated function in the NCBI nr database. All annotated metagenome sequence assemblies (Celera/PGA) discussed in the current manuscript are available through the DOE-JGI IMG/M (Markowitz et al., 2012) website (http://img.jgi.doe.gov/m) under IMG taxon OID numbers as follows: YNPSite06 (2022920004/2013515000), Site07 (2022920013/2014031006), Site15 (2022920016/2015219002), Site16 (2022920018/2016842003), Site05 (2022920003/2013954000), Site20 (2022920020/2016842008), and Site17 (2022920021/2016842005).

### Ribosomal RNA sequence analyses

All bacterial 16S rRNA sequences from the 16S rRNA-specific PCR clone libraries were aligned and screened for chimeras with Bellerophon (Huber et al., 2004) with subsequent manual curation. OTUs were determined using the CAP3 assembler (Huang and Madan, 1999) at the 99% demarcation level. Rarefaction curves were determined, and the Chao1 and ACE richness indexes and the Fisher’s alpha, Shannon-Weaver, and Simpson’s diversity indexes were calculated for each library (EcoSim version 7.0, Gotelli and Entsminger, 2001; EstimateS v. 8.0, Colwell, 2009). The RDP Bayesian Classifier (Wang et al., 2007) was used to assign taxonomy to 16S rRNA sequences at the 80% confidence level (Figures 5B and 6B), and all sequences belonging to the Chloroflexi were aligned with reference sequences corresponding to *Escherichia coli* positions 29–1349 (1321 positions). Alignments were masked with bacterial complexity filters in ARB (Ludwig et al., 2004). A phylogenetic tree was produced using the BioNJ algorithm (Gascuel, 1997) (Figure 2) and bootstrapped with 1000 replicates. Reference sequences shorter than the initial alignment were subsequently added to the tree using the ARB parsimony tool. Consensus maximum-likelihood trees were produced from 1000 replicate trees using RaxML (Stamatakis, 2006). A maximum-likelihood tree based upon amino acid alignments of PscD sequences was constructed using PhyML (Guindon et al., 2010).

### Statistical analyses

A distance matrix of environmental variables was constructed by calculating Gower coefficients using the R statistical environment (R Development Core Team, 2012). The Gower coefficient allows for different data types (qualitative presence/absence vs. quantitative numerical) with different dimensional scales to be combined into a general dissimilarity metric (Gower, 1971). Geochemical variables were treated as factors and were correlated to this distance matrix using the envfit function of the vegan package (Oksanen et al., 2012). Metagenomic scaffolds larger than 10 kbp were subjected to analysis using oligonucleotide composition. All possible tri-, tetra-, penta-, and hexanucleotides were counted with custom perl scripts, and normalized to the length of the scaffold. Normalized oligonucleotide composition matrices were subjected to k-means clustering with a range of k = 4–12 with 100 trials each. Clusters were reported when at least 10 scaffolds grouped together in 90% or greater Monte-Carlo simulations. The composite summary of these k-means trials was displayed as an interaction network using the program Cytoscape 2.8.1 (Shannon et al., 2003).

### Broad functional analysis of metagenome sequences

Assembled sequence from each of the phototrophic sites was annotated as described in Inskeep et al. (2010) and predicted proteins from the scaffolds were assigned TIGRFAM protein families (Selengut et al., 2007) using HMMER 3 (Eddy, 2011) with *e*-value cutoff of 1e−6. PCA and statistical analysis of site group differences was performed using the STAMP v2.0 software (Parks and Beiko, 2010). The White’s non-parametric *T*-test and ANOVA tests were used to test for differences between two site groups and multiple site groups respectively. Two-way clustering was performed using row-standardized (across sites) average TIGRFAM category abundance data using the Euclidean distance metric and complete-linkage hierarchical clustering in MeV 4.8 (Saeed et al., 2003) software. Other details regarding TIGRFAM analysis are described in this issue (Inskeep et al., 2013).

## Conflict of Interest Statement

The authors declare that the research was conducted in the absence of any commercial or financial relationships that could be construed as a potential conflict of interest.

## Appendix

**Table A1:** **Community diversity estimated from 16S sequence libraries**.

Site_N umber	N	99% OTUs	Singletons	ACE	Chao1	SD	95U	95D	alpha	SD	Shannon	Simpson
BLVA_5	305	69	32	120.9	96.6	12.9	134.8	80.5	27.8	2.5	3.2	10.4
BLVA_20	364	130	102	773.9	873.1	318.3	1791.1	462.5	72.3	6.0	3.8	14.1
White Creek_6	367	69	34	131.3	141.3	36.6	253.3	97.3	25.1	2.1	3.4	19.2
Chocolate Pots_7	380	127	100	402	421.1	94.0	669.1	286.6	66.9	5.4	3.4	9.2
Mushroom Spring _15 (Bacteria)	314	112	66	214.1	220.9	37.7	322.5	168.3	62.3	5.6	4.1	37.5
Mushroom Spring _15 (Archaea)	265	35	16	65	77.7	33.2	199.5	46.1	10.8	1.1	2.7	8.9
Fairy Geyser_16	360	137	96	390.3	408.1	87.5	639.4	283.2	80.7	6.8	4.1	32.8

Richness indexes ACE, Chao1 (w/95% confidence intervals) and diversity indexes (Fisher’s alpha, Shannon-Weaver, and Simpson’s Index).
